# Interleukin-10 produced by B cells is crucial for the suppression of Th17/Th1 responses, induction of T regulatory type 1 cells and reduction of collagen-induced arthritis

**DOI:** 10.1186/ar3736

**Published:** 2012-02-08

**Authors:** Natalie A Carter, Elizabeth C Rosser, Claudia Mauri

**Affiliations:** 1 Centre for Rheumatology Research, Division of Medicine, University College London, Rayne Building, 5 University Street, London, WC1E 6JF, UK

## Abstract

**Introduction:**

Interleukin-10 (IL-10) producing B cells, also known as regulatory B (Breg) cells, play a key role in controlling autoimmunity. Our laboratory and others have demonstrated a pivotal role for Bregs in rheumatological disorders, including experimental models of arthritis and lupus. The aim of this study was to identify the role of endogenous IL-10 secreting B cells *in vivo *in controlling the induction and disease progression of collagen-induced arthritis (CIA).

**Methods:**

We generated chimeric mice that had IL-10 knocked-out specifically in the B cell population. These mice were compared with wild-type (WT) B cell chimeric mice for their susceptibility to CIA.

**Results:**

Here we report that chimeric mice specifically lacking IL-10 producing B cells (IL-10^-/- ^B cell) developed an exacerbated CIA compared to chimeric wild type B cell (WT B cell) mice. A marked increase in inflammatory Th1 and Th17 cells were detected in IL-10^-/-^B cell mice compared to WT B cell mice. Furthermore, there was a reduction in IL-10 secreting CD4^+ ^Tr1 cells in these animals.

**Conclusions:**

IL-10 producing B cells restrain inflammation by promoting differentiation of immuno-regulatory over pro-inflammatory T cells and, hence, act to maintain tolerance.

## Introduction

CIA-induced joint destruction is widely accepted to develop as a result of the secretion of pro-inflammatory Th1 cytokines, such as IFNγ and IL-12 [1-3]. These Th1 cytokines facilitate the infiltration of neutrophils and macrophages into the joint, which stimulates the production of both TNFα and IL-1 that ultimately results in joint destruction and pannus formation [4,5]. In addition to this, CIA is mediated by pathogenic B cells, which produce anti-collagen antibodies that are indicative of disease development [5] and can induce arthritis upon transfer [6,7]. This taken together with the fact that B cell deficient mice (μMT) are resistant to CIA [8] shows that CIA is both a T and B cell-mediated disease.

The role of IL-10 has been well documented in experimental arthritis [9-13] and other autoimmune disorders [14-18]. It has been shown that CIA is exacerbated in IL-10 deficient DBA mice [12], although the relevant contributions of IL-10 secreted by T cells and B cells cannot be revealed using IL-10^-/- ^animals. The importance of B cell derived IL-10 in CIA has been confirmed by previous work in this laboratory [9,10]. Several regulatory B cell subsets have now been identified and most share the release of IL-10 as a common mechanism of action. In experimental arthritis, we have shown that the transfer of the main producers of IL-10, namely CD19^+^CD21^hi^CD23^hi^CD1d^hi ^transitional 2 marginal zone precursor B cells (T2-MZP), prevents or ameliorates established disease [9,19]. Similarly, transfer of CD5^+^CD1d^hi ^B cells (B10) controls the development of the contact hypersensitivity response (CHS) [20]. In each instance, Bregs isolated from IL-10 deficient mice (IL-10^-/-^) mice failed to suppress the development of autoimmune diseases [21-25]. In order to assess the importance of all subsets of IL-10 secreting regulatory B cells, we generated chimeric mice that lack IL-10 specifically on all B cells. Thus, providing us with a unique environment to assess the role of B cell derived IL-10 in joint inflammation.

Previous work in this laboratory has shown a pivotal role for endogenous B cell-derived IL-10 in the context of antigen induced arthritis (AIA) [19]. AIA is induced by immunization with mBSA emulsified in Complete Freunds Adjuvant (CFA), followed a week later by intra-articular injection with mBSA [26]. The incidence of disease (that is, antigen-mediated joint swelling) is 100% and the disease is characterized by acute inflammation which is resolved within one month [27]. In the latter stages of disease, anti-mBSA antibodies are also produced [28], hence, this model incorporates both the DTH response and the development of an autoimmune-like disease. IL-10^-/- ^B cell mice have an exacerbated AIA arthritis phenotype, including increased clinical scores and knee swelling, enhanced Th17 and Th1 development and a reduction in regulatory T cells [19].

Next we wanted to elucidate and validate the role of IL-10 secreting Bregs in CIA, a polyarthritis model involving both severe inflammation and cartilage and bone erosion. CIA differs from AIA in several key areas. CIA cannot be induced in B cell deficient mice, whereas AIA is a predominantly T- cell mediated disease that can be induced in B cell deficient mice that develop an exacerbated AIA [8,19]. Additionally, different genetic backgrounds and modes of immunizations are commonly used. The courses of these diseases are also significantly different. AIA is a monoarthritis, which can be resolved in under one month, whereas CIA can take several months to develop and can go into remission in one or more paws.

In this paper, we have shown that in animals lacking IL-10 specifically on their B cells, T cell differentiation is skewed to pro-inflammatory Th1 and Th17 subtypes, at the expense of the differentiation and maintenance of immune-regulatory Tr1 cells. These conditions result in exacerbated experimental arthritis in IL-10^-/- ^B cell mice as compared to WT B cell mice.

## Materials and methods

### Financial disclosure

This work and NC is funded by Arthritis Research UK http://www.arthritisresearchuk.org/arthritis_research.aspx by the programme grant to CM (MP/17707) and by the equipment grant (19367) to CM and NC. ER is funded by ARUK PhD studentship (NE/PhD/19607) to CM. The funders had no role in study design, data collection and analysis, decision to publish, or preparation of the manuscript.

### Ethics statement

These studies have been reviewed and approved by the Home Office U.K. This work was conducted under UK Home Office Project Licence number PPL 70/7108.

### Animals and antibodies

IL-10^-/- ^and μMT animals on the H2^q ^background were generated by backcrossing the original IL-10/H2^b ^and μMT/H2^b ^with DBA/1 H2^q ^mice. The mice were typed by PCR, and IL-10 KO^-/-^H2^q ^were further backcrossed into DBA/1. Mice from the 15^th ^generation (DBA/1IL-10 KO^-/-^) were used for experiments. All animals were bred and maintained under specific pathogen-free conditions at the animal facility at University College London, UK. All antibodies were purchased from BD Biosciences, Oxford, UK.

### Generation of chimeric mice

Chimeric mice were generated as previously published [17]. Briefly, recipient μMt mice received 800 cGy of γ-irradiation via a caesium source. Five hours following irradiation recipients received 2 × 10^6 ^donor bone marrow cells. Bone marrow preparations were depleted of T cells by negative selection with a MACS magnetic column (Miltenyi Biotech, Bergisch Gladbach, Germany). To generate mice where the absence of IL-10 was exclusively restricted to B cells, μMT mice were reconstituted with a mixture of bone marrow consisting of 80% from μMT (no B cell differentiation) with 20% from IL-10^-/- ^mice. Control mice received 80% from μMT and 20% bone marrow from WT mice (to give a normal B cell compartment). Two additional control groups were included: 100% of bone marrow from μMT into WT recipients (control for the absence of B cells) or 80% WT and 20% IL-10^-/- ^bone marrow into μMT recipients (this will assess the effect of 20% reduction in IL-10 production by non-B cell lymphocytes in the response observed). Chimeras were left to fully reconstitute their peripheral lymphoid system over at least eight weeks before use in CIA experiments. The absence of B cells (CD19-expressing splenocytes) in the group that received 100% μMT bone marrow confirmed the total ablation of the host bone marrow by irradiation. In contrast, the three other groups showed numbers of CD19^+ ^B cells and CD4^+^T cells equivalent to numbers found in non chimeric WT B6 mice.

### Induction and assessment of collagen-induced arthritis and histology of joints

Male DBA/1 mice were immunized with 100 μg of type II bovine collagen (CII) emulsified in CFA (Diffco Laboratories, Oxford, UK) as previously described [9]. The development of arthritis was assessed daily for the duration of the experiment. The clinical severity of arthritis was graded as follows: 0, normal; 1, slight swelling and/or erythema; 2, pronounced edematous swelling; 3, pronounced edematous swelling plus joint rigidity; and 4, laxity. Each limb was graded, allowing a maximal clinical score of 16 for each animal. All clinical evaluations were performed in a blinded manner. All the mice are kept in accordance with the local guidelines.

Hind paws were removed post-mortem and fixed in 10% (w/v) buffered formalin and decalcified in 5% EDTA. After decalcification, the paraffin sections were stained with hematoxylin-eosin. Two independent observers evaluated the slides histologically. The slides were graded as: 0, normal, no damage; 1, minimal synovitis, cartilage loss, and bone erosion limited to discrete foci; 2, synovitis and erosion present, but normal joint architecture intact; and 3, extensive erosion and joint architecture disrupted.

### Serum anti-collagen antibody levels

Anti-CII Abs were determined as previously described [29]. Briefly, microplates (Nunc) were coated with (2 μg/ml) bovine CII overnight, blocked with 2% BSA and then incubated with serial dilutions of the testing sera. Bound IgG1 and IgG2a were detected by incubation with alkaline phosphatase-conjugated sheep anti-mouse IgG1 and IgG2a respectively (The Binding Site, Schwetzingen, Germany) followed by TMB (Sigma, St Louis, MO, USA).

### Flow cytometric analysis of intracellular cytokine synthesis

Intracellular cytokine analysis was performed as previously described [10]. Briefly, inguinal lymph node cultures were suspended at 5 × 10^5 ^cells/ml in complete medium with PMA (50 ng/ml Sigma-Aldrich, St Louis, MO, USA), ionomycin (500 ng/ml Sigma-Aldrich, St Louis, MO, USA) and GolgiPlug (BD Biosciences, Oxford, UK) for five hours. Cells were then stained with extracellular markers, followed by permeabilization and incubation with anti-mouse IL-10 APC, IFNγ APC or IL-17 PE mAbs. The cells were acquired with a BD LSR flow cytometer (BD Biosciences, Oxford, UK) and analysed using FlowJo software.

### In vivo cytokine capture assay

This was carried out using BD *in vivo *IFNγ and IL-2 capture assay kits. Briefly, mice were injected intraperitoneally with 10 μg of NA/LE biotin-conjugated anti-mouse IFNγ antibody in 200 μL in sterile PBS. Blood samples were collected from injected mice after 12 hours, and serum was isolated. Serum cytokine levels were then analyzed using an ELISA based method.

### Cytokine secretion assay

Lymph node cultures were suspended at 5 × 10^5 ^cells/ml in complete medium with anti-CD3 (1 μg/ml). After incubation at 37°C for 48 hours, the plates were centrifuged; the supernatants were collected and stored at - 80°C until further analysis. Cytokine concentration in the supernatants was determined using mouse Th1/Th2 cytokine FlowCytomix kit (Bender Medsystems, AachenGermany), following the manufacturer's instructions.

### Regulatory T cell suppression assay/proliferation assay

Spleens were removed post-mortem and CD4^+^T cells, Treg and Teff cells were negatively isolated. Cells were cultured for 60 hours with either complete medium or with anti-CD3 (1 μg/ml). Cultures were then pulsed overnight with 1 μCi of (H^3) ^thymidine, harvested and counted in a scintillation counter (LKB Instruments, Mt Waverley, Victoria, Australia).

### Statistical analysis

For the statistical analysis of the data, the Mann-Whitney *U *test and the Fisher exact test were applied to analyze clinical results. Unpaired *t *tests were applied in all other experiments. *P *< 0.05 was considered significantly different.

## Results

### IL-10 is essential for the regulation of experimental arthritis

It is well documented that mice lacking IL-10 have a predisposition to immune-driven colitis and inflammation of the gut [16,30,31]. Furthermore, it has been shown that CIA develops with increased incidence and severity in IL-10 deficient animals [12,13]. Unlike the wild type animals, the IL-10^-/- ^animals do not enter remission (when inflammation has subsided) and, as such, joint swelling and redness does not permanently recede. In order to unravel the relative contribution of endogenous B cell derived IL-10 in a polyarthritis model, we generated mixed bone marrow chimera mice with IL-10 knocked-out specifically on B cells. As a control we generated bone marrow chimera mice with a normal WT B cell compartment. We have previously published that both WT B cell and IL-10^-/- ^B cell animals show no major differences in either T or B cell phenotypes either in naïve or immunized animals (Additional file 1[19]). Furthermore, the WT B cell chimeric mice developed CIA with the same incidence and severity as WT mice that have not undergone any irradiation procedures.

**Figure 1:**
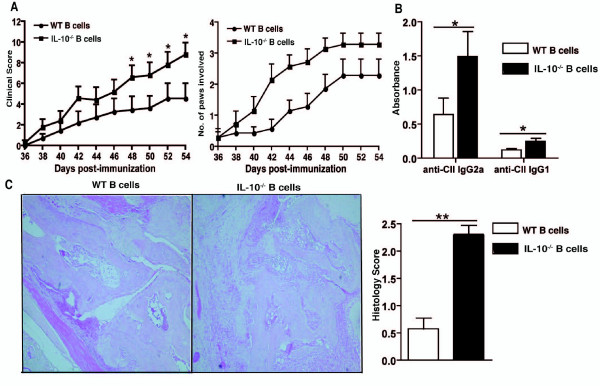
**Lack of endogenous B cell-derived IL-10 results in exacerbated CIA**. **A**. Chimeric mice were generated that lacked IL-10 specifically on B cells. Mean clinical score for CIA and numbers of diseased paws are shown. Data are expressed as mean ± SEM (*n *= 20, cumulative data from four separate experiments with *n *= 5 per experimental group). Data were compared by statistical analysis using the Fisher test. * *P *< 0.05. **B**. On Day 45 following immunization, mice were bled and the serum levels of total IgG1 and IgG2a CII-specific IgG antibodies were measured by ELISA. Data are expressed as mean ± SEM (*n *= 20, cumulative data from four separate experiments with *n *= 5 per experimental group). Data were compared by statistical analysis using the unpaired t test. * *P *< 0.05. **C**. The gradation of arthritis was scored from 0 to 4 according to the intensity of lining layer hyperplasia, mononuclear cell infiltration and pannus formation. Data are expressed as mean ± SEM (*n *= 20, cumulative data from four separate experiments with *n *= 5 per experimental group). Representative histological sections are also shown.

The CIA clinical scores and number of affected paws for IL-10^-/- ^B cell mice as compared to the WT B cell mice were significantly increased (Figure 1A). In addition to this, IL-10^-/- ^B cell mice produce increased anti-collagen antibodies, especially of the pathogenic IgG2a class (Figure 1B). The histological analysis revealed an exacerbated disease in IL-10^-/- ^B cell mice as shown by increased cellular infiltration and the loss of joint architecture in affected paws of IL-10^-/- ^B cell chimeric mice compared to WT B cell mice (Figure 1C). The majority of the joints from the IL-10^-/- ^B cell chimeric mice group were severely damaged, exhibiting proliferation of the pannus and accumulation of inflammatory cells, in contrast the WT B cell group the displayed minimal thickening of the cartilage and cellular infiltration (Figure 1C).

### Pro-inflammatory cytokines IFNγ and IL-17 are increased in arthritic IL-10^-/- ^B cell animals

**Figure 2:**
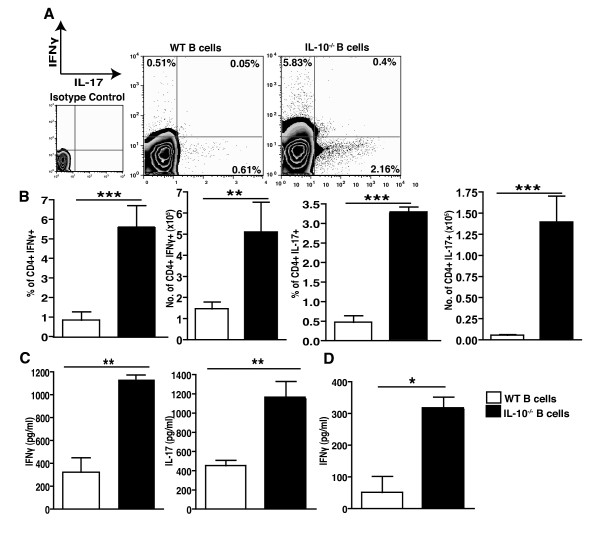
**Lack of B cell-derived IL-10 results in increased Th17 and Th1 responses**. **A**. A total of 35 days after CIA induction, draining lymph node cells were excised and cultured with PMA plus ionomycin in the presence of Brefeldin A for five hours. The intracellular levels of IFNγ and IL-17 were measured. Dot plots are gated on the CD4^+ ^population. **B**. Numbers indicate percentages or absolute numbers of cells in the quadrants. Data show mean ± SEM (*n *= 4), representative of four experiments. Data were compared by statistical analysis using the unpaired *t *test. *** *P *< 0.001. **C**. Supernatant was collected from lymph node cells stimulated *in vitro *for 48 hours with anti-CD3, and then secreted IFNγ and IL-17 was measured by cytokine FlowCytomix kit. Data show mean ± SEM (*n *= 4), representative of four experiments. Data were compared by statistical analysis using the unpaired *t *test. ** *P *< 0.01. **D**. Additionally, a BD *in vivo *cytokine capture assay was used to assess *in vivo *levels of IFNγ over a 12-hour period. The mean levels of IFNγ-conjugated to antibody in serum are shown ± SEM (*n *= 5), representative of two separate experiments. Data were compared by statistical analysis using the unpaired *t *test. * *P *< 0.05.

Pathogenesis of arthritis is very dependent upon the secretion of pro-inflammatory cytokines and the subsequent recruitment of inflammatory cells [32]. IL-17 and IFNγ are both potent pro-inflammatory cytokines that recruit T cells and macrophages to the site of inflammation resulting in both inflammation and joint destruction, key indicators of arthritic disease. Using intracellular cytokine staining it was demonstrated that the IL-10^-/- ^B cell mice with arthritis had an increased CD4^+ ^IFNγ producing population (Figure 2A, B). We found increased levels of Th1 cells in the LN of arthritic IL-10^-/- ^B cell mice compared to WT B cell mice at days 12, 35 and 45 post-immunization with collagen (Additional file 2). Moreover, there was a major increase in secreted IFNγ from IL-10^-/- ^B cell mice as compared to WT B cell mice, as observed in LN culture supernatant (Figure 2C). To confirm that IL-10^-/-^B cell mice present with dysregulated IFNγ production *in vivo*, we administered biotin-anti-IFNγ-labelled antibodies. Mice were bled 12 hours later and serum levels of cytokine-biotin-anti-cytokine mAb complexes were determined by ELISA [33]. The results in Figure 2D demonstrate that during the acute phase of inflammation a significant increase of circulating IFNγ in IL-10^-/-^B cell compared to WT B cell mice was reported, revealing a mechanism employed by Bregs to control disease.

Additionally, investigation of IL-17 levels clearly demonstrated an increase in IL-17 production, as seen by both multiplex bead array and IL-17 intracellular staining, in the IL-10^-/- ^B cell mice (Figure 2A-C). Th17 cells were only significantly increased in the LN, but not the spleen, of arthritic IL-10^-/- ^B cell mice compared to WT B cell mice at days 12, 35 and 45 post-immunization with collagen (Additional file 2 and data not shown). Therefore, in addition to IFNγ, IL-17 could also play an important role in both the increased inflammation and tissue destruction seen in the IL-10^-/- ^B cell mice. However, the percentage and number of CD4^+ ^IFNγ^+ ^IL-17^+ ^double producing cells was not significantly different in the WT B cell and IL-10^-/- ^B cell mice (data not shown).

### Regulatory T cells are reduced in IL-10^-/- ^B cell animals

**Figure 3:**
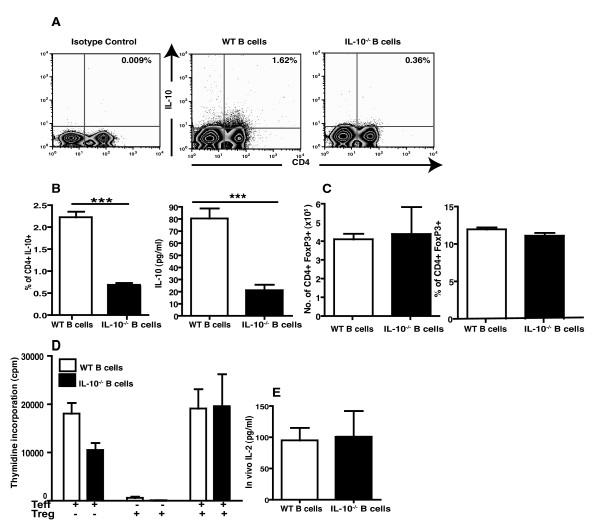
**Lack of B cell-derived IL-10 results in decreased Tr1 responses, however FoxP3^+ ^Tregs are unaffected**. **A**. A total of 35 days after CIA induction, draining lymph node cells were excised and cultured with PMA plus ionomycin in the presence of Brefeldin A for five hours. The CD4+ T cell population was identified using anti-CD4 FITC mAb and intracellular levels of IL-10 were measured. **B**. Numbers indicate percentages of cells in the quadrants. Data shown mean ± SEM (*n *= 4), are representative of four experiments. Data were compared by statistical analysis using the unpaired *t *test. *** *P *< 0.001. In addition, supernatant was collected from lymph node cells stimulated *in vitro *for 48 hours with anti-CD3, and then secreted IL-10 was measured by cytokine FlowCytomix kit. Data show mean ± SEM (*n *= 4), and are representative of four experiments. Data were compared by statistical analysis using the unpaired *t *test. *** *P *< 0.001. **C**. Tregs were assessed using anti-CD25, anti-CD4 and anti-FoxP3 mAb. Numbers indicate percentages of CD4 gated FoxP3^+ ^cells or the absolute number of cells in a draining lymph node. Data show mean ± SEM (*n *= 4), and are representative of four experiments. **D**. Spleens were taken from IL-10^-/- ^B cell and WT B cell animals and used to isolate CD25+ Treg cells using Milteyi Biotech magnetic beads. T-effector cells were isolated from immunized WT B6 mice using the same process. The cells were cultured for 72 hours with anti-CD3 antibody (1 μg/ml). For 12 hours before harvesting cells were pulsed with (^3^H) thymidine. Data shown are mean ± SEM of triplicate wells and are representative of two independent experiments. **E**. Additionally, a BD *in vivo *cytokine capture assay was used to assess *in vivo *levels of IL-2 over a 12-hour period. The mean levels of IL-2-conjugated to antibody in serum are shown ± SEM (*n *= 4), are representative of two separate experiments. Data were compared by statistical analysis using the unpaired *t *test.

Analysis of CD4^+ ^derived IL-10 by flow cytometry showed that the IL-10^-/- ^B cell mice have a decreased capacity to secrete IL-10 from their T cell population (Figure 3A, B). This was demonstrated at an early time point (Day 12 post- immunization) and during CIA disease (Day 35) and 45 days post-immunization (Additional file 2). This reduction in IL-10 secretion was also confirmed using multiplex bead array on LN culture supernatants (Figure 3B). Interestingly, this reduction in CD4^+ ^T cell derived IL-10 is only observed in the LN cells and not in splenocytes. The reduction in CD4^+ ^T cell derived IL-10 is of particular interest as it suggests that B-cell-derived IL-10 is essential for the development of these anti-inflammatory CD4^+ ^IL-10^+ ^cells. This clearly corroborates previous data showing that IL-10 secreting T2-MZP Bregs have the ability to promote Tr1 cell development *in vitro *[19,34].

It is interesting to note that FoxP3^+^Treg numbers are not reduced in the LN of IL-10^-/- ^B cell mice with CIA (Figure 3C). We did not see any significant differences in FoxP3^+^Treg numbers in the LN of IL-10^-/- ^B cell mice compared to WT B cell mice on days 12, 35 or 45 post-immunization with collagen (Additional file 2). We also compared FoxP3^+^Treg numbers in the spleen and were unable to see any differences in IL-10^-/- ^B cell mice compared to WT B cell mice with CIA (data not shown). Moreover, neither the suppressive function of these Tregs (Figure 3D) nor *in vivo *levels of IL-2 (Figure 3E) were modulated in these animals. We have previously published that during the development of antigen-induced arthritis in IL-10^-/- ^B cell mice we can see a decrease in FoxP3^+ ^Treg numbers and expression of FoxP3 specifically at the site of inflammation (both the synovial membrane of the affected knee and the inguinal LN draining that knee) [19].

We did not see differences in number or function of FoxP3^+ ^Tregs during CIA development in IL-10^-/- ^B cell mice. It is important to note that splenocytes and lymph node cells from these IL-10^-/- ^B cell animals do have normal proliferative responses to anti-CD3 (Additional file 1B). Taken together these data suggest that FoxP3^- ^CD4^+ ^IL-10 secreting Tr1 population is preferentially affected by B cell- derived IL-10 during CIA.

## Discussion

The importance of IL-10 in disease control has been clearly demonstrated by the use of IL-10^-/- ^animals. We and others have shown that immune mediated colitis [16,30], EAE [15] and experimental arthritis [12] are exacerbated in these animals. However, this does not resolve the question of which IL-10 producing cell types are able to control inflammation.

Our results take advantage of chimeric animals that lack IL-10 specifically in their B cells. These animals had increased pro-inflammatory cytokines and antibodies in circulation, firmly establishing the importance of B cell derived IL-10 in regulating disease (Figures 1B and 2).

In a colitis model, TCR^-/- ^μMT mice develop a more severe disease than an only-TCR^-/- ^mouse, indicating that B cells are as important as T cells in this inflammatory disease [35]. Interestingly, the IL-10^-/- ^B cell mice developed a "colitis-like" disease with symptoms including rectal pro-lapse with some bleeding, sticky stool consistency, increased intestinal-gas (seen by dissection) and loss of body weight (data not shown). This supports the idea that B cell derived IL-10 is an important component of the hierarchy that regulates and suppresses the immune system, a concept that has been proved numerous times in transfer experiments [9,17] and disease induction in μMT mice [35,36]. It even suggests that in certain inflammatory models IL-10 secreting Bregs can be apical to regulatory T cells in prevention of autoimmunity and the maintenance of tolerance [37].

It is well established that IL-10 producing Tr1 cells control the expansion of Teff cells and reduce the production of proinflammatory cytokines *in vitro *[38-42]. However, to date there is a scarcity of information about the stimuli promoting *in vivo *differentiation of Tr1 and whether they are promoted by other cells. Here, our data confirm and expand upon the importance of Bregs in the differentiation and maintenance of Tr1 cells *in vivo *in the context of chronic disorders [19,34,43]. Therefore, taking into account our data and those already available in the literature, it is feasible to speculate that IL-10 producing B cells may control the regulatory hierarchy, including the proper development of anti-inflammatory T cells, leading to the maintenance of tolerance. This combined information reveals a conflicting hypothesis to research that attributes Tregs as the most important component of immune regulation [44], and that B cells only have a pathogenic role in autoimmune disease [45].

Of interest, unlike in AIA where animals display a reduction in both the number of FoxP3^+ ^Tregs and their expression of FoxP3^+ ^in IL-10^-/- ^B cell mice compared to WT B cell mice, the number of FoxP3^+ ^Tregs and FoxP3^+ ^expression were similar in both groups during CIA. The discrepancy between the results in these two models could be due to several reasons. The course of arthritis is very different in the two diseases. Mice immunized with collagen in CFA develop disease three-to-four weeks post-immunization, whereas mice with AIA are assessed five days post immunization. In addition, it is possible to obtain a sufficient number of cells for flow cytometry analysis from the synovia (obtained from the knee) in mice developing AIA, we were unable to assess the frequencies of Tregs in the synovia of mice with CIA. Thus, timing and location could account for the differential data on Tregs in the IL-10^-/-^B cell mice in the two models. Nevertheless, in both an acute inflammatory model and a systemic experimental arthritis, IL-10^-/- ^B cell mice have an exacerbated arthritis phenotype.

## Conclusions

These data shed some light on the mechanism of action of IL-10- secreting regulatory B cells. We have shown that in IL-10^-/- ^B cell mice T cell differentiation is skewed to pro-inflammatory Th1 and Th17 subtypes, whereas regulatory Tr1 cells are reduced as compared to WT B cell animals (Figures 2 and 3A, B). These increased inflammatory conditions result in exacerbated arthritis in IL-10^-/- ^B cell mice as compared to WT B cell mice (Figure 1). These data confirm previous findings from this laboratory, and others, establishing the power of B cell produced IL-10 in maintenance of tolerance and prevention of multiple experimental autoimmune diseases [9,10,19,20,34,43,46,47].

## Abbreviations

AIA: antigen-induced arthritis; Breg: regulatory B cells; CFA: complete Freund's adjuvant; CHS: contact hypersensitivity response; CIA: collagen-induced arthritis; DTH: delayed type hypersensitivity; EAE: experimental autoimmune encephalomyelitis; FoxP3: forkhead box P3; IFNγ: interferon gamma; IL: interleukin; LN: lymph node; PMA: phorbol 12-myristate 13-acetate; T2-MZP: transitional type 2-marginal zone precursor B cells; Teff: effector T cell; Th1: T helper 1 cells; Th17: T helper 17 cells; TNFα: tumor necrosis factor alpha; Tr1: T regulatory type 1 cells; Treg: regulatory T cell; WT: wild type.

## Competing interests

The authors declare that they have no competing interests.

## Authors' contributions

NC generated the chimeric mice, completed the experimental work, analyzed the data, designed and coordinated the experiments, and wrote the manuscript. ER assisted with flow cytometry and ELISA experimental work and helped to score the animals and histological samples. CM conceived the study, participated in its design and coordination, and wrote the manuscript. All authors read and approved the final manuscript.

## Supplementary Material

Additional file 1 **Supplemental Data 1**. Data to demonstrate that B and T cell development and T_eff _functional responses were unaffected in IL-10^-/- ^B cell animals. Click here for file

Additional file 2 **Supplemental Data 2**. Data showing the percentage of FoxP3^+ ^Tregs and CD4- derived IL-10, IFNγ and IL-17 on days 12, 35 and 45 post-immunization for CIA. Click here for file
