# Supplementary material for: Interleukin-10 produced by B cells is crucial for the suppression of Th17/Th1 responses, induction of T regulatory type 1 cells and reduction of collagen-induced arthritis
Source: Arthritis Res Ther. 2012 Feb 8;14(1):R32. doi: 10.1186/ar3736 (PMC3392827; doi:10.1186/ar3736)
Supplement: Additional file 1 — Supplemental Data 1. Data to demonstrate that B and T cell development and Teff functional responses were unaffected in IL-10-/- B cell animals. [file ar3736-S1.PDF]

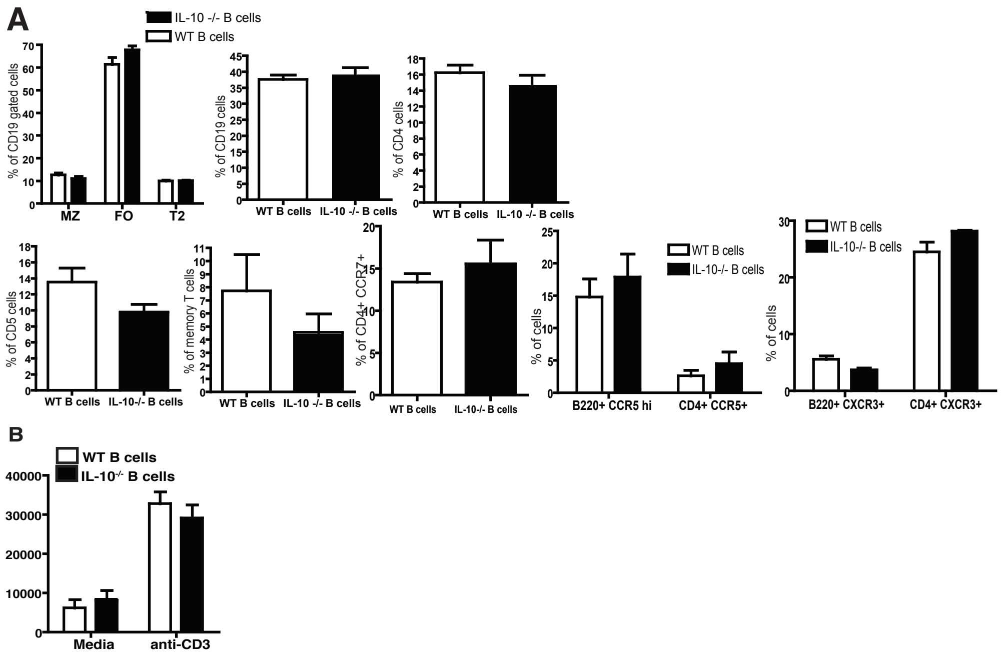

Supplemental Data 1:

A. B and T cell development was unaffected in IL-10$^{-/-}$ B cell animals

FACS analysis was used to identify B cell subsets, CD4^{+} T cells, CD5^{+} B cells, memory T cells, CCR7 expressing CD4^{+} T cells, CCR5 expressing B and CD4^{+} T cells and CXCR3 expressing B and CD4^{+} T cells. Dots plots were created for each population and numbers indicate percentages of cells in the quadrants. Data show mean ± SEM of 5 individual mice in each group, and are representative of three experiments. Data were compared by statistical analysis using the unpaired t test and shown to be not significant.

B. Teff functional responses are normal in mice that lack B cell-derived IL-10

CD4^{+} cells were isolated from lymph nodes by negative selection using Milteyi Biotech magnetic beads and then cultured with either medium alone or anti-CD3 antibody (1µg/ml) for 48 hours. For 12 hours before harvesting cells were pulsed with [^{3}H] thymidine. Data shown are mean ± SEM of triplicate wells and are representative of 4 experiments.
