# Supplementary material for: Interleukin-10 produced by B cells is crucial for the suppression of Th17/Th1 responses, induction of T regulatory type 1 cells and reduction of collagen-induced arthritis
Source: Arthritis Res Ther. 2012 Feb 8;14(1):R32. doi: 10.1186/ar3736 (PMC3392827; doi:10.1186/ar3736)
Supplement: Additional file 2 — Supplemental Data 2. Data showing the percentage of FoxP3+ Tregs and CD4- derived IL-10, IFNγ and IL-17 on days 12, 35 and 45 post-immunization for CIA. [file ar3736-S2.PDF]

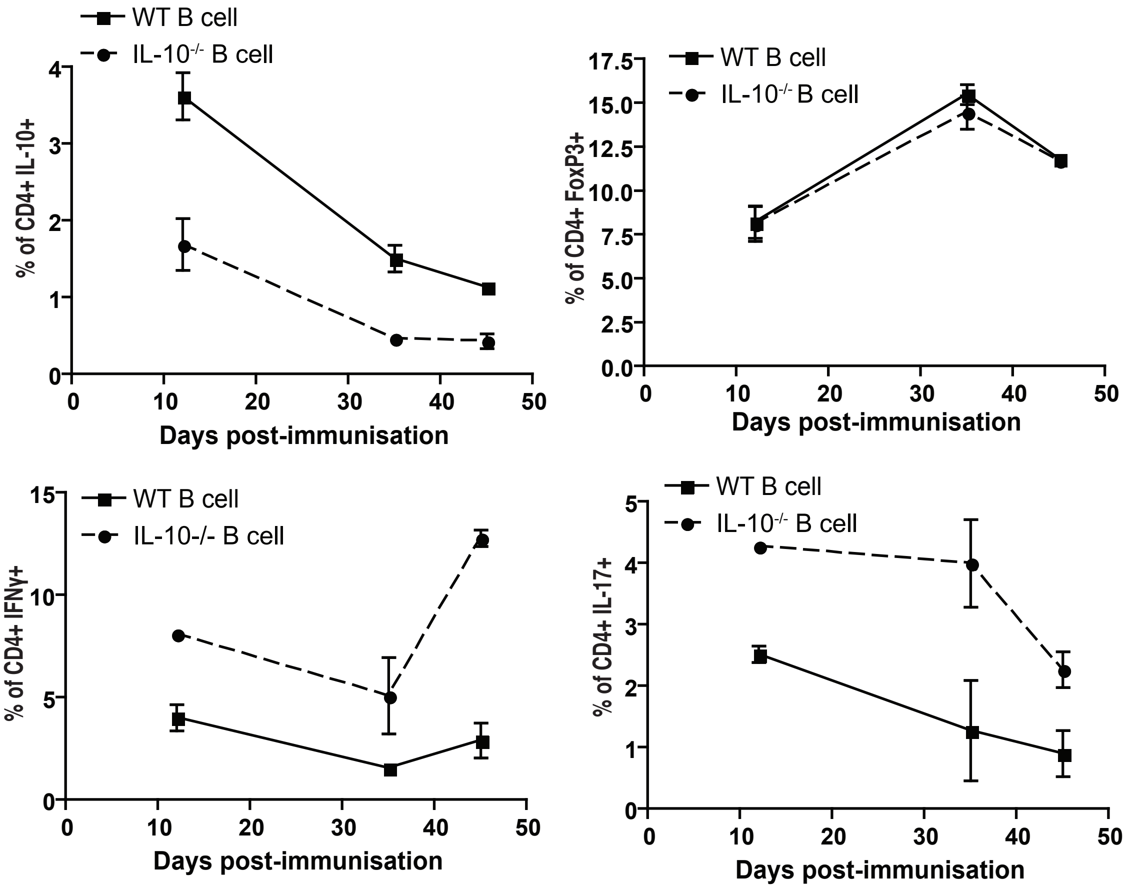

Supplemental Data 2:
12, 35 or 45 days after CIA induction, draining lymph node cells were excised and cells were cultured with PMA plus ionomycin in the presence of Brefeldin A for 5 hours. The graph show the intracellular levels of IL-10, IFNγ and IL-17 measured at different time point after arthritis induction. The percentage of FoxP3$^+$CD4$^+$T cells was measured directly ex vivo (without any stimulation). Dot plots are gated on the CD4$^+$ T cells. Numbers indicate percentages of cells in the quadrants. Data show mean ± SEM (n=4).
